# Age, time from injury to surgery and quadriceps strength affect the risk of revision surgery after primary ACL reconstruction

**DOI:** 10.1007/s00167-021-06517-8

**Published:** 2021-03-04

**Authors:** Riccardo Cristiani, Magnus Forssblad, Gunnar Edman, Karl Eriksson, Anders Stålman

**Affiliations:** 1 Department of Molecular Medicine and Surgery, Stockholm Sports Trauma Research Center, Karolinska Institutet, Stockholm, Sweden; 2 Capio Artro Clinic, FIFA Medical Centre of Excellence, Sophiahemmet Hospital, Valhallavägen 91, 11486 Stockholm, Sweden; 3 Department of Orthopaedics, Stockholm South Hospital, Karolinska Institutet, Stockholm, Sweden

**Keywords:** Anterior cruciate ligament, ACL, Revision, Limb symmetry index, Graft, Quadriceps strength, Meniscus, Age, Muscle strength

## Abstract

**Purpose:**

To identify preoperative, intraoperative and postoperative factors associated with revision anterior cruciate ligament reconstruction (ACLR) within 2 years of primary ACLR.

**Methods:**

Patients who underwent primary ACLR at our institution, from January 2005 to March 2017, were identified. The primary outcome was the occurrence of revision ACLR within 2 years of primary ACLR. Univariate and multivariate logistic regression analyses were used to evaluate preoperative [age, gender, body mass index (BMI), time from injury to surgery, pre-injury Tegner activity level], intraoperative [graft type, graft diameter, medial meniscus (MM) and lateral meniscus (LM) resection or repair, cartilage injury] and postoperative [side-to-side (STS) anterior laxity, limb symmetry index (LSI) for quadriceps and hamstring strength and single-leg-hop test performance at 6 months] risk factors for revision ACLR.

**Results:**

A total of 6,510 primary ACLRs were included. The overall incidence of revision ACLR within 2 years was 2.5%. Univariate analysis showed that age < 25 years, BMI < 25 kg/m^2^, time from injury to surgery < 12 months, pre-injury Tegner activity level ≥ 6, LM repair, STS laxity > 5 mm, quadriceps strength and single-leg-hop test LSI of ≥ 90% increased the odds; whereas, MM resection and the presence of a cartilage injury reduced the odds of revision ACLR. Multivariate analysis revealed that revision ACLR was significantly related only to age < 25 years (OR 6.25; 95% CI 3.57–11.11; *P* < 0.001), time from injury to surgery < 12 months (OR 2.27; 95% CI 1.25–4.17; *P* = 0.007) and quadriceps strength LSI of ≥ 90% (OR 1.70; 95% CI 1.16–2.49; *P* = 0.006).

**Conclusion:**

Age < 25 years, time from injury to surgery < 12 months and 6-month quadriceps strength LSI of ≥ 90% increased the odds of revision ACLR within 2 years of primary ACLR. Understanding the risk factors for revision ACLR has important implications when it comes to the appropriate counseling for primary ACLR. In this study, a large spectrum of potential risk factors for revision ACLR was analyzed in a large cohort. Advising patients regarding the results of an ACLR should also include potential risk factors for revision surgery.

**Level of evidence:**

III.

## Introduction

The number of anterior cruciate ligament (ACL) reconstructions has increased significantly in recent years [20]. Primary ACL reconstruction (ACLR) has been shown to be successful in restoring knee laxity and improving subjective knee function [9]. However, graft failure after primary ACLR remains a serious event for patients, often requiring revision surgery and repeating the long rehabilitation process [40]. In addition, revision ACLR is associated with poorer patient-reported outcome measures compared with primary ACLR [4, 10, 18].

Some patient- and surgery-related risk factors for revision ACLR have been suggested in previous studies. Younger age has been consistently associated with an increased risk of revision ACLR [1, 21, 23, 26, 32, 36]. However, contrasting results regarding the effect on the risk of revision surgery by other variables, such as gender, time from injury to primary ACLR, graft type and diameter, have been reported by several authors [1, 2, 11, 17, 23, 24, 30, 31, 36]. In addition, current knowledge regarding the effect of knee laxity and muscle strength measurements after primary ACLR on the risk of revision ACLR is limited.

There is a need for a comprehensive and detailed analysis of preoperative, intraoperative and postoperative risk factors for revision ACLR. An awareness of the effect of multiple factors on the risk of revision ACLR could help clinicians to counsel patients undergoing primary ACLR about this complication. In addition, knowledge of potentially modifiable risk factors for revision ACLR might be used to target these factors and reduce the risk of this serious event. Previous studies have shown that graft rupture and revision ACLR occur most frequently within the first 2 years of primary ACLR [8, 21]. The purpose of this study was to identify preoperative, intraoperative and postoperative factors associated with revision ACLR within 2 years of primary ACLR, in a large cohort. It was hypothesized that younger age, female gender, hamstring tendon (HT) autograft, concomitant meniscus resection at the time of primary ACLR, abnormal anterior laxity (side-to-side [STS] difference > 5 mm) and asymmetrical (limb symmetry index [LSI] of < 90%) quadriceps and hamstring strength 6 months after primary ACLR would be risk factors for revision ACLR within 2 years of primary surgery.

## Materials and methods

Patient data were extracted from our clinic database. Patients registered for primary ACLR from January 2, 2005, to March 7, 2017 were assessed for eligibility. Only patients who underwent primary ACLR with an HT or a bone–patellar tendon–bone (BPTB) autograft and had no concomitant ligament injuries were included. Patients who underwent bilateral ACLR only contributed their index knee for analysis.

### Surgical technique and rehabilitation

All patients underwent surgery using a single-bundle autologous HT or BPTB technique. The graft was chosen according to the surgeon’s preference. For the ACLRs performed with the HT graft, the triple or quadruple semitendinosus tendon or semitendinosus and gracilis tendons were used. The BPTB graft was harvested as the central third of the patellar tendon with two bone blocks. The femoral tunnel was drilled using an anteromedial portal technique. In the majority of cases, the grafts were fixed using an EndoButton fixation device (Smith & Nephew, Andover, MA) or, for the BPTB graft, an interference screw on the femoral side and No. 2 Ethibond sutures (Ethicon, Somerville, NJ) tied over an AO bi-cortical screw with a washer as a post or using an interference screw on the tibial side. Meniscal repair was performed with an arthroscopic all-inside technique using a Fast-Fix suture anchor device (Smith & Nephew) for tears located in the dorsal and middle portion of the menisci. Tears located in the anterior portion of the menisci were repaired using an outside-in technique with No. 0 PDS (Ethicon).

All the patients followed a standardized postoperative rehabilitation protocol. In the event of an isolated ACLR or an ACLR with concomitant meniscus resection, full weight-bearing and full range of motion were encouraged as tolerated. If meniscal repair was performed, patients wore a postoperative hinged knee brace for 6 weeks. Flexion was limited from 0° to 30° for the first 2 weeks after surgery, from 0° to 60° for the third and fourth weeks and from 0° to 90° for the 5th and 6th weeks. From the 7th week, the knee brace was discontinued and progressive weight-bearing was allowed. For all patients, quadriceps strengthening was restricted to closed kinetic chain exercises during the first 3 months. On the basis of muscle strength (quadriceps and hamstring strength LSI of ≥ 90%), co-ordination and functional performance (single-leg-hop test LSI of ≥ 90%), patients were allowed to return to sport 6 months postoperatively at the earliest.

### Arthrometric evaluation

Patients underwent an instrumented laxity assessment 6 months after surgery. All knee laxity evaluations were performed at our outpatient clinic by experienced sports medicine physiotherapists, using the KT-1000 arthrometer (MEDmetric, San Diego, CA). A standard 30-lb force, corresponding to a 134-N anterior tibial load, at 20° of knee flexion, was applied. At least 3 measurements of each knee were made and the median value was registered. The postoperative difference in displacement (STS difference) between the ACL-reconstructed knee and the healthy knee was expressed in millimeters.

### Isokinetic strength and single-leg-hop test performance assessment

Isokinetic strength and single-leg-hop test performance were assessed using a standardized protocol 6 months postoperatively.

Isokinetic concentric quadriceps and hamstring strength were measured bilaterally at 90°/s using the Biodex System 3 (Biodex Medical Systems, Shirley, New York, USA). The test was performed in a range of motion between 90° and 10° of knee flexion, always starting with the contralateral uninjured knee. Prior to the test, the patients warmed up using a stationary cycling ergometer at low resistance for 10 min. Patients were given a verbal description of the test and two to three practical trials were allowed before testing. Each patient performed five maximum quadriceps and hamstring contractions with each leg. The patients were encouraged verbally during the test. The peak quadriceps and hamstring torque values (highest achieved values) were registered.

The single-leg-hop test [7] was used to assess functional hop performance. The test was performed with the patient standing on one leg and being instructed to jump straight ahead as far as possible and land on the same leg. The test was considered successful if the landing was stable. If the patient landed with an early touchdown of the contralateral limb, had a loss of balance or took additional hops after landing, the hop was repeated. Patients were initially given a verbal description of the test and they were allowed to perform practical trials until they felt confident about the test. Three trials were performed for each leg, always starting with the contralateral uninjured leg. The best trial for each leg was registered.

### Data sources

Several potential risk factors for revision ACLR were investigated. Data were collected in our clinic registry. *Preoperative factors* included age, gender, body mass index (BMI), time from injury to surgery and pre-injury Tegner activity level [33]. For the purpose of the study, age was dichotomized into unbiased classes close to the median (< 25 years or ≥ 25 years). The dichotomization of the BMI at 25 kg/m^2^ was selected because patients with a BMI of ≥ 25 kg/m^2^ are classified as overweight [39]. The time from injury to surgery was also dichotomized into unbiased classes close to the median (< 12 months or ≥ 12 months). Finally, the pre-injury Tegner activity level was classified as high (≥ 6) or low (< 6). The *intraoperative factors* that were evaluated were graft type (HT or BPTB autograft), graft diameter for HT autograft (< 8 mm or ≥ 8 mm), medial meniscus (MM) resection, MM repair, lateral meniscus (LM) resection, LM repair and the presence of a cartilage injury. The *postoperative factors* (6 months) that were included were instrumented laxity (KT-1000 arthrometer) measurements, isokinetic quadriceps and hamstring strength and single-leg-hop test performance. Knee laxity was classified according to the International Knee Documentation Committee examination form [15]. Abnormal laxity was defined as an STS difference greater than 5 mm (IKDC grades C and D). The results of the isokinetic quadriceps and hamstring strength tests and single-leg-hop test were classified based on the limb symmetry index (LSI) as symmetrical (LSI ≥ 90%) or asymmetrical (LSI < 90%) for each test [7, 14, 35].

### Outcome

The outcome of this study was the occurrence of revision ACLR within 2 years of primary ACLR. Patients who underwent revision ACLR at our institution or other institutions in the country were identified through their unique Swedish personal identity number [22] in the Swedish National Knee Ligament Registry [34]. Patients were followed for 2 years (730 days) after primary ACLR. Those who underwent revision ACLR performed during this time frame were identified. Ethical approval for this study was obtained from the regional ethics committee, Karolinska Institutet (Dnr 2016/1613–31/2).

### Statistical analysis

The Statistical Package for Social Sciences, SPSS (Version 25.0, IBM Corp., Armonk, New York, USA), was used for the statistical analysis. All the variables were summarized with standard descriptive statistics such as frequency, mean and standard deviation. Univariate logistic regression analyses were performed with age (< 25 years vs ≥ 25 years), gender, BMI (< 25 kg/m^2^ vs ≥ 25 kg/m^2^), time from injury to surgery (< 12 months vs ≥ 12 months), pre-injury Tegner activity level (high ≥ 6 vs low < 6), graft (HT vs BPTB autograft), HT graft diameter (< 8 mm vs ≥ 8 mm), MM resection, MM repair, LM resection, LM repair, cartilage injury, 6-month STS laxity (> 5 mm vs ≤ 5 mm) and quadriceps and hamstring strength and single-leg-hop test performance (LSI ≥ 90% vs LSI < 90%) as independent variables, with revision ACLR as the dependent variable. A multivariate logistic regression analysis was used to determine independent risk factors for revision ACLR. Only variables attaining a significant *P* value in the univariate analysis were entered in the multivariate analysis. BMI and the single-leg-hop test were excluded from the multivariate regression model owing to missing data. The drop-outs for these variables did not match and the collapsed drop-out rate for these two variables was large. All relationships were expressed as odds ratios (OR) with 95% confidence intervals (CI). The level of significance in all analyses was 5% (two tailed).

## Results

A total of 6,510 patients who underwent primary ACLR were included. Of these, 166 patients (2.5%) underwent revision ACLR during the 2-year follow-up. Patient characteristics for the no-revision ACLR (*n* = 6,344) and revision ACLR (*n* = 166) groups are summarized in Table 1.

**Table 1:** Patient characteristics and factors associated with the risk of revision ACLR in univariate logistic regression analysis

	No-revision ACLR	Revision ACLR		
	(*n* = 6344)	(*n* = 166)	OR (95% CI)	*P* value
*Preoperative factors*				
Age at surgery, years, mean ± SD	28.7 ± 10.8	21.2 ± 7.7		
Age < 25 years	2837 (44.7)	137 (82.5)	5.88 (4.00–9.09)	< 0.001
Age ≥ 25 years	3507 (55.3)	29 (17.5)		
Gender				
Female	2825 (44.5)	75 (45.2)	1.02 (0.75–1.39)	n.s
Male	3519 (55.5)	91 (54.8)		
BMI, kg/m^2^, mean ± SD	24.3 ± 3.5	23.2 ± 2.6		
< 25	3357 (70.3)	124 (87.3)	2.94 (1.78–5.00)	< 0.001
≥ 25	1417 (29.7)	18 (12.7)		
	*n* = 4774	*n* = 142		
Time from injury to surgery, months, mean ± SD	16.6 ± 30.5	8.4 ± 15.6		
< 12 months	4232 (71.2)	142 (88.2)	3.03 (1.89–5.00)	< 0.001
≥ 12 months	1712 (28.8)	19 (11.8)		
	*n* = 5944	*n* = 161		
Pre-injury Tegner activity level, median (range)	7 (1–10)	8 (1–10)		
High, ≥ 6	4798 (85.8)	144 (93.5)	2.39 (1.25–4.55)	0.008
Low, < 6	796 (14.2)	10 (6.5)		
	*n* = 5594	*n* = 154		
*Intraoperative factors*				
Graft type				
HT autograft	5908 (93.1)	155 (93.4)	1.04 (0.56–1.93)	n.s
BPTB autograft	436 (6.9)	11 (6.6)		
Graft diameter (for HT autograft), mean ± SD	8.2 ± 1.6	8.4 ± 0.7		
< 8 mm	1088 (22.4)	28 (20.4)	0.89 (0.58–1.37)	n.s
≥ 8 mm	3771 (77.6)	109 (79.6)		
	*n* = 4859	*n* = 137		
Medial meniscus surgery				
Resection	974 (15.4)	16 (9.6)	0.59 (0.35–0.98)	0.04
Repair	419 (6.6)	6 (3.6)	0.53 (0.23—1.20)	n.s
Lateral meniscus surgery				
Resection	1036 (16.3)	33 (19.9)	1.27 (0.86–1.87)	n.s
Repair	255 (4.0)	15 (9.0)	2.37 (1.37–4.09)	0.002
Cartilage injury				
Yes	1141 (18.0)	16 (9.6)	0.48 (0.28–0.81)	0.007
No	5203 (82.0)	150 (90.4)		
*Postoperative factors (6 months)*				
KT-1000 STS difference, mm ± SD	1.7 ± 2.2	2.7 ± 2.4		
> 5 mm	250 (4.9)	16 (11.7)	2.59 (1.51–4.41)	0.001
≤ 5 mm	4887 (95.1)	121 (88.3)		
	*n* = 5137	*n* = 137		
Isokinetic quadriceps strength LSI, mean ± SD	84.4 ± 16.3	84.8 ± 16.6		
≥ 90%	1836 (33.7)	70 (54.7)	2.38 (1.67–3.38)	< 0.001
< 90%	3620 (66.3)	58 (45.3)		
	*n* = 5456	*n* = 128		
Isokinetic hamstring strength LSI, mean ± SD	90.0 ± 18.9	89.7 ± 14.2		
≥ 90%	2500 (45.9)	62 (48.4)	1.10 (0.78–1.57)	n.s
< 90%	2949 (54.1)	66 (51.6)		
	*n* = 5449	*n* = 128		
Single leg hop test LSI, mean ± SD	92.4 ± 14.0	96.9 ± 8.5		
≥ 90%	3120 (67.7)	91 (81.3)	2.07 (1.28–3.34)	0.003
< 90%	1489 (32.3)	21 (18.7)		
	*n* = 4609	*n* = 112		

Data are reported as *n* (%), unless otherwise indicated

*ACLR*, anterior cruciate ligament; *BMI*, body mass index; *BPTB*, bone-patellar tendon-bone; *CI*, confidence intervals; *HT*, hamstring tendons; *LSI*, limb symmetry index; *OR*, odds ratio; *SD*, standard deviation; *STS*, side-to-side

### Univariate analyses

Univariate logistic regression analysis showed that younger age (< 25 years) (OR 5.88; 95% CI 4.00–9.09; *P* < 0.001), BMI < 25 kg/m^2^ (OR 2.94; 95% CI 1.78–5.00; *P* < 0.001), time from injury to surgery < 12 months (OR 3.03; 95% CI 1.89–5.00; *P* < 0.001), pre-injury Tegner activity level ≥ 6 (OR 2.39; 95% CI 1.25–4.55; *P* = 0.008), LM repair (OR 2.37; 95% CI 1.37–4.09; *P* = 0.002) and 6-month postoperative KT-1000 STS difference > 5 mm (OR 2.59; 95% CI 1.51–4.41; *P* = 0.001), quadriceps strength LSI of ≥ 90% (OR 2.38; 95% CI 1.67–3.38; *P* < 0.001) and single-leg-hop test LSI of ≥ 90% (OR 2.07; 95% CI 1.28–3.34; *P* = 0.003) increased the odds of revision ACLR, whereas MM resection (OR 0.59; 95% CI 0.35–0.98; *P* = 0.04) and the presence of a cartilage injury (OR 0.48; 95% CI 0.28–0.81; *P* = 0.007) reduced the odds. No significant correlation was found between revision ACLR and female gender, HT graft, graft diameter (for HT autograft), MM repair, LM resection and 6-month hamstring strength LSI of ≥ 90% (Table 1).

### Multivariate analyses

Multivariate logistic regression analysis (total patients included: 4,423 no-revision ACLR, 115 revision ACLR) showed that younger age (< 25 years) (OR 6.25; 95% CI 3.57–11.11; *P* < 0.001), time from injury to surgery < 12 months (OR 2.27; 95% CI 1.25–4.17; *P* = 0.007) and 6-month quadriceps strength LSI of ≥ 90% (OR 1.70; 95% CI 1.16–2.49; *P* = 0.006) increased the odds of revision ACLR. No significant correlation was found between revision ACLR and pre-injury Tegner activity level ≥ 6, MM resection, LM repair, cartilage injury and 6-month KT-1000 STS difference > 5 mm (Table 2).

**Table 2:** Factors associated with the risk of revision ACLR in multivariate logistic regression analysis

	SE	OR (95% CI)	*P* value
Preoperative factors			
Age < 25 years	0.29	6.25 (3.57–11.11)	< 0.001
Time from injury to surgery < 12 months	0.30	2.27 (1.25–4.17)	0.007
Pre-injury Tegner activity level ≥ 6	0.46	1.94 (0.78–4.83)	n.s
Intraoperative factors			
Medial meniscus resection	0.33	1.10 (0.57–2.12)	n.s
Lateral meniscus repair	0.34	1.69 (0.85–3.32)	n.s
Cartilage injury	0.30	1.09 (0.59–1.99)	n.s
Postoperative factors (6 months)			
KT-1000 STS difference > 5 mm	0.21	1.33 (0.87–2.04)	n.s
Isokinetic quadriceps strength LSI ≥ 90%	0.19	1.70 (1.16–2.49)	0.006

*ACLR*, anterior cruciate ligament reconstruction; *CI*, confidence interval; *HT*, hamstring tendons; *LSI*, limb symmetry index; *OR*, odds ratio; *SE*, standard error; *STS*, side-to-side

## Discussion

The most important findings in this study were that patient age < 25 years, time from injury to primary ACLR < 12 months and 6-month quadriceps strength LSI of ≥ 90% increased the odds of revision ACLR within 2 years of primary ACLR. The overall incidence of revision ACLR within 2 years was 2.5% in the entire cohort.

In the literature, younger age has consistently been associated with an increased risk of revision ACLR [1, 21, 23, 26, 36]. This consistent finding might be secondary to a higher post-surgery activity level [21] and a greater likelihood of returning to pivoting sports for younger patients [28, 37]. In addition, younger patients may be less tolerant of recurrent instability after graft failure and more willing to undergo revision surgery if they are unable to return to their previous activity level [24, 29].

In this study, gender had no effect with respect to the risk of revision ACLR. These results support those of previous studies [1, 16, 23, 26, 36, 41], showing that gender is probably not related per se to the incidence of revision ACLR.

Another discussed risk factor for revision ACLR is the timing of primary ACLR. Previous large cohort registry studies investigated the effect of time from injury to primary ACLR on the risk of revision ACLR within 2 years. However, they reported conflicting results. Andernord et al. [1] stratified their cohort in several time from injury to surgery intervals and found that there were no significant differences in the incidence of revision surgery. On the other hand, Snaebjörnsson et al. [31] reported that patients undergoing primary ACLR within 3 months ran a significantly higher risk of revision ACLR. It should be noted that both these studies did not consider the patient’s pre-injury activity level. It has been shown that patients with a higher pre-injury activity level tend to undergo early ACLR [3] and this might bias the results toward a higher risk of revision with earlier ACLR. In the present study, a higher pre-injury activity level was significantly related to a higher risk of revision ACLR in the univariate analysis, but its effect disappeared in the multivariate regression model. It is possible that, after ACLR, patients do not always maintain the same pre-injury activity level. On the contrary, a time from injury to primary ACLR of < 12 months was found to be an independent factor that increased the odds of revision ACLR. As reported by Andernord et al. [1], it might be hypothesized that patients undergoing early primary ACLR are more prone to undergo early revision ACLR as well, which would then be detected within the 2-year follow-up and bias the results towards a lower risk of revision ACLR with delayed ACLR [1]. Another hypothesis might be that patients with a longer time from injury to surgery interval adapt to an injured knee, reducing the risk of exposing their knee to risk activities for graft failure and subsequent revision ACLR [1, 31].

We were unable to find any correlation between medial or lateral meniscus resection or repair at the time of primary ACLR and the risk of undergoing revision ACLR. The significant correlation between LM repair and revision ACLR in the univariate analysis disappeared in the multivariate analysis. It has been shown that younger age strongly increases the odds of LM repair [6]. There might be a willingness on the part of the surgeon to attempt to repair and save the meniscus whenever possible in younger patients. Younger age was the most important factor affecting the odds of revision ACLR in the multivariate regression model. This suggests that LM repair is not per se a significant risk factor for revision ACLR, but the important factor is the age at the time of primary ACLR. The same consideration could be applied to a cartilage injury, which was found to be a factor that reduced the odds of revision ACLR in the univariate analysis but not in the multivariate regression model. Older age is significantly associated with the presence of a cartilage injury at the time of primary ACLR [6]. Again, it appears that a cartilage injury per se is not a risk factor for revision ACLR, while age at primary ACLR is a strong risk factor. These results are in line with those of a recent large cohort study [31], based on the Swedish and Norwegian knee ligament registries, which found that a cartilage injury at the time of primary ACLR does not affect the risk of undergoing revision ACLR within 2 years.

The selection of an HT autograft over a BPTB autograft was not a risk factor for revision surgery in the present study. These results contrast with those of recent large cohort registry studies, which found an increased risk of revision ACLR with HT autografts compared with BPTB autografts [11, 26, 27]. These contrasting findings might be related to the fact that, at our institution, patients received a BPTB autograft most likely because they were considered at a higher risk of graft failure and revision surgery. On the other hand, national registry studies include patients undergoing ACLR at several institutions and some of them may perform ACLR almost exclusively with HT autografts.

There is an ongoing debate about the effect of the diameter of HT autografts as a risk factor for revision ACLR. Biomechanical studies have shown a correlation between graft size and ultimate failure load [13]. However, in clinical studies, some authors found higher rates of revision surgery with HT graft diameters less than 8 mm [23, 25]; whereas, others [1, 17, 38] did not find any correlation between graft diameter and the risk of revision surgery. Inderhaug et al. [17] reported that the use of smaller graft diameters (< 8 mm) does not result in a higher risk of revision. Our results are in line with the latter study. We found that the diameter of the HT graft (< 8 mm vs. ≥ 8 mm) did not affect the risk of revision ACLR.

The significant association between an STS laxity difference of > 5 mm 6 months after primary ACLR and revision ACLR in the univariate analysis disappeared in the multivariate regression model. It has been shown that younger patients have increased odds of having a STS laxity difference of > 5 mm 6 months after primary ACLR [5]. Younger age was the most important factor affecting the odds of revision ACLR in the adjusted analysis. This might suggest that postoperative STS laxity may only have a minor effect on the risk of revision ACLR and that the age at the time of primary ACLR is more important.

One unanticipated result was that a symmetrical (LSI of ≥ 90%) isokinetic quadriceps strength 6 months after primary ACLR was an independent risk factor for revision ACLR. Previous studies reported that muscular asymmetries (LSI of < 90%) are risk factors for ACL graft tears and knee re-injuries [12, 19]. One possible explanation of this finding might be that, at our institution, an important discharge criterion for allowing patients to return to pivoting activities was the achievement of a quadriceps strength LSI of ≥ 90% at the 6-month follow-up after primary ACLR. Patients who did not achieve this result were recommended not to return to pivoting activities, to continue with rehabilitation and to repeat the isokinetic strength assessment some two to three months later. This could have biased the results towards a higher risk of revision ACLR with a symmetrical (LSI of ≥ 90%) quadriceps strength 6 months after primary ACLR. Patients achieving a quadriceps strength LSI of ≥ 90% at this time point may have returned to sport and pivoting activities earlier, exposing their knee to graft failure and subsequent revision ACLR.

The main strength of this study is the analysis of a large cohort (6510 patients) with the inclusion of a relatively large number of patients (*n* = 166) who underwent revision ACLR. Patients underwent surgery and had their postoperative assessment at the same institution. Rehabilitation and recommendations for return to sport and pre-injury activities were standardized. These characteristics make this study different from previous studies based on national registries which included patients undergoing surgery at different clinics with different surgical techniques, rehabilitation protocols and non-standardized recommendations for return to sport and pre-injury activities. The large and varied study cohort, in terms of preoperative, intraoperative and postoperative potential risk factors studied, makes the results highly generalizable. Finally, one important strength was the comprehensive evaluation of several risk factors for revision ACLR. This large cohort study simultaneously investigated different preoperative, intraoperative and postoperative risk factors for revision ACLR in a large cohort.

There are several limitations. The outcome of this study was the occurrence of revision ACLR. It is known that, for a variety of reasons, not all patients with graft failure choose to undergo revision ACLR. So, the incidence of graft failure is probably higher than that of revision ACLR and the risk factors for graft failure might be different from those for revision ACLR. Another possible limitation is the relatively short follow-up. The outcome of this study was the occurrence of revision ACLR within 2 years. Revisions are known to occur even after this follow-up period. However, over time and in a real-life setting, there could be many other factors that possibly increase or decrease the risk of revision ACLR, complicating the interpretation of the risk factors for revision ACLR. Missing data for some risk factors (BMI and single-leg-hop test) associated with revision ACLR in the univariate analysis prevented their inclusion in the multivariate regression model. This might have affected the results. However, the large sample size and the inclusion of several other risk factors for revision ACLR probably mitigated this limitation. Finally, other factors that might affect the risk of graft failure and revision ACLR, such as surgeon experience, graft tunnel location, precise timing and criteria for return to sport and post-surgery activity level, have not been controlled for. Our registry does not contain this information.

## Conclusion

Age < 25 years, time from injury to surgery < 12 months and 6-month quadriceps strength LSI of ≥ 90% increased the odds of revision ACLR within 2 years of primary ACLR. Understanding the risk factors for revision ACLR has important implications when it comes to the appropriate counseling for primary ACLR. In this study, a large spectrum of potential risk factors for revision ACLR was analyzed in a large cohort. Advising patients regarding the results of an ACLR should also include potential risk factors for revision surgery.
